# Epidemiology and outcome of *Clostridium difficile* infections in patients hospitalized in Internal Medicine: findings from the nationwide FADOI-PRACTICE study

**DOI:** 10.1186/s12879-016-1961-9

**Published:** 2016-11-08

**Authors:** Giorgio Cioni, Pierluigi Viale, Stefania Frasson, Francesco Cipollini, Francesco Menichetti, Nicola Petrosillo, Sergio Brunati, Patrizia Spigaglia, Chiara Vismara, Alessandra Bielli, Fabrizio Barbanti, Giancarlo Landini, Grazia Panigada, Gualberto Gussoni, Erminio Bonizzoni, Giovanni Pietro Gesu, for the Research Department of FADOI, A. Costantino, R. Masciari, G. Amato, A. Fontanella, F. Gallucci, L. Improta, M. R. Poggiano, P. Rabitti, G. Uomo, G. Civardi, M. Confalonieri, M. Grandi, C. Sacchetti, T. Baldini, G. Cioni, S. Miglioli, M. Sarti, M. P. Landini, D. Panuccio, E. Romboli, C. Vocale, F. Berti, L. Bonito, M. L. Gozzo, D. Manfellotto, S. Natili, A. Patti, P. Piccolo, E. Pistella, C. Santini, R. Pastorelli, M. Pellegrinotti, C. P. Quaratino, R. Bona, P. Gnerre, F. Lillo, L. Parodi, A. Bovero, L. Santoriello, F. Agnelli, F. Colombo, G. Gesu, G. Lombardi, V. Lanzetti, S. Biagiotti, P. Clerici, A. Mazzone, G. Brusco, L. Magnani, S. Tirella, C. Terulla, E. Manso, C. Nitti, A. Salvi, T. Ciarambino, C. Pepe, C. Politi, R. Frediani, A. Moschella, L. Lodolo, G. Barasolo, M. C. Bertoncelli, F. Milano, M. Campanini, G. L. Molinari, S. Pittau, A. De Bernochi, M. Giusti, G. Montegrosso, M. Errico, F. Mastroianni, L. Tauro, A. Caddori, C. G. Prasciolu, C. Di Naso, M. Romano, A. D’Angelo, L. Genco, G. Mangano, F. Arena, C. Biagioni, B. Cimolato, G. Landini, C. Nozzoli, A. Poli, G. Rossolini, F. Burzigotti, S. Francioni, S. Lenti, I. A. Galanti, C. Belcari, B. Longo, D. Salamone, I. Chiti, P. Lencioni, G. Panigada, L. Teghini, M. Gambacorta, U. Perrotta, G. Battaglia, E. Pizzato, A. Vian, E. De Menis, G. Bordignon, P. Tramontin, C. Doroldi, P. Ravagnan, P. Sartore, M. Leoni, P. Pauletto, R. Rigoli, C. Callegari, A. Sacchetta, A. Vendrame

**Affiliations:** 1 Department of Internal Medicine, Pavullo nel Frignano Hospital, Modena, Italy; 2 Infectious Diseases Unit, Teaching Hospital “Policlinico S. Orsola Malpighi”, Alma Mater Studiorum University of Bologna, Bologna, Italy; 3 Clinical Research Department, FADOI Foundation, Piazzale L. Cadorna, 15, Milan, Italy; 4 Internal Medicine, Hospital “Vittorio Emanuele II”, Amandola Ascoli Piceno, Italy; 5 Infectious Diseases Unit, University Hospital of Pisa, Pisa, Italy; 6 2nd Infectious Diseases Division, National Institute for Infectious Diseases, “Lazzaro Spallanzani” IRCCS, Rome, Italy; 7 Department of Internal Medicine, Abbiategrasso Hospital, Milan, Italy; 8 Department of Infectious, Parasitic and Immune-mediated Diseases, Istituto Superiore di Sanità, Rome, Italy; 9 Clinical Chemistry and Microbiology Laboratory, Niguarda Ca’ Granda Hospital, Milan, Italy; 10 Department of Internal Medicine, “Santa Maria Nuova” Hospital, Florence, Italy; 11 Department of Internal Medicine, “S.S. Cosma e Damiano” Hospital, Pescia Pistoia, Italy; 12 Section of Medical Statistics and Biometry “GA Maccacaro”, Department of Clinical Science and Community, University of Milan, Milan, Italy

**Keywords:** *Clostridium difficile*, Internal Medicine, Incidence, Predictors, Outcome

## Abstract

**Background:**

*Clostridium difficile* (CD) is a leading cause of diarrhoea among hospitalized patients. The objective of this study was to evaluate the rate, the optimal diagnostic work-up, and outcome of CD infections (CDI) in Internal Medicine (IM) wards in Italy.

**Methods:**

PRACTICE is an observational prospective study, involving 40 IM Units and evaluating all consecutive patients hospitalized during a 4-month period. CDI were defined in case of diarrhoea when both enzyme immunoassay for GDH, and test for A/B toxin were positive. Patients with CDI were followed-up for recurrences for 4 weeks after the end of therapy.

**Results:**

Among the 10,780 patients observed, 103 (0.96 %) showed CDI, at admission or during hospitalization. A positive history for CD, antibiotics in the previous 4 weeks, recent hospitalization, female gender and age were significantly associated with CDI (multivariable analysis). In-hospital mortality was 16.5 % in CD group *vs* 6.7 % in No-CD group (*p* < 0.001), whereas median length of hospital stay was 16 (IQR = 13) *vs* 8 (IQR = 8) days (*p* < 0.001) among patients with or without CDI, respectively. Rate of CD recurrences was 14.6 %. As a post-hoc evaluation, 23 out of 34 GDH+/Tox- samples were toxin positive, when analysed by molecular method (a real-time PCR assay). The overall CD incidence rate was 5.3/10,000 patient-days.

**Conclusions:**

Our results confirm the severity of CDI in medical wards, showing high in-hospital mortality, prolonged hospitalization and frequent short-term recurrences. Further, our survey supports a 2–3 step algorithm for CD diagnosis: EIA for detecting GDH, A and B toxin, followed by a molecular method in case of toxin-negative samples.

**Electronic supplementary material:**

The online version of this article (doi:10.1186/s12879-016-1961-9) contains supplementary material, which is available to authorized users.

## Background

*Clostridium difficile (CD)* is a Gram-positive, spore-forming, exotoxin-producing anaerobic bacillus responsible for a range of clinical conditions, from asymptomatic infection to slight diarrhoea, pseudomembranous colitis, toxic megacolon and bowel perforation [1].

Clinical suspicion and appropriate and timely laboratory diagnosis are crucial for the treatment and prevention of *Clostridium difficile* infection *(CDI)*. Although a number of laboratory tests are available for the diagnosis of CDI, including direct detection of CD products [2], the optimal diagnostic strategy is still debated. A two- or three-stage algorithm may improve the sensitivity of CDI diagnosis [2, 3], but a great heterogeneity exists among laboratories as for implementation of these procedures which are affected by availability of resources and expertise.

Elderly patients are at the highest risk of infection, especially if they are hospitalized or residents in nursing care home. Suggested risk factors for developing CDI include prior antibiotic use, acid suppressive agents [4, 5], previous CDI [6], malignancies, gastrointestinal disorders [7] and inflammatory bowel diseases [8]. Recurrent CDI is associated with significant morbidity, mortality and additional need of healthcare resources. Recurrences generally occur within 4 weeks from the end of antibiotic therapy, with reported incidence of 20–30 % [9, 10].

Rates of CDI have been increasing since 2000 in both North America and Europe [11], with the highest incidence rates in elderly patients [12]. This increased prevalence of CDI has prompted the development of national infection prevention programs. According to a survey published in 2014, around half of European countries had issued a national guideline for CDI prevention [13]. The United Kingdom requires public reporting of CDI cases by individual hospitals, while in Germany severe cases of CDI must be reported to government health authorities. Further, different options of CDI surveillance for acute care hospitals have been tested across Europe [14]. In Italy, no nationwide program for surveillance of CDI has been implemented to date, and there is a lack of reliable data on the epidemiology of CDI at national level. Published information on the burden of CDI in Italy comes from a single hospital or a small group of hospitals, is often retrospective and the reported incidence is highly variable [15–19]. This variability may be related in part to heterogeneity in diagnostic methods used from one laboratory to the next. Therefore, a nationwide prospective survey is needed to better evaluate the incidence of CDI among Italian hospitals, also applying a standardized diagnostic method, and especially in medical wards which account for the majority of reported cases [17, 20].

To address this need, the Italian Scientific Society of Hospital Internal Medicine (FADOI), in cooperation with the Italian Association of Clinical Microbiologists (AMCLI), planned, coordinated and implemented the prospective national surveillance program “FADOI-PRACTICE”, to determine the incidence of CDI in Internal Medicine units in Italy and collect data on patient outcomes, recurrences, and CDI risk factors.

## Methods

### Patients and methods

The FADOI-PRACTICE is an observational, prospective, multicentre study involving 40 hospitals in Italy, aimed at evaluating the incidence of CDI (new cases developed at least 3 days after admission to hospital/10,000 patients-days) and risk factors for CD in Internal Medicine units (IMU). Moreover, the study allowed to evaluate the prevalence of CDI in IMUs (cases of diarrhoea at hospital admission/study population), the length of hospital stay and all-cause in-hospital mortality in the groups of patients with and without CDI. Among patients with CDI, the percentage of CD recurrences, either as in-hospital cases and during post-discharge follow-up (within 4 weeks after the end of CDI therapy), as well as the rate of re-hospitalization and all-cause mortality during follow-up were also assessed. Recurrences were defined as episodes occurring after resolution of symptoms (i.e. 3 days free from diarrhoea) and completion of the cycle of therapy for CDI [1, 2].

The study enrolled all consecutive patients hospitalized in an IMU for any cause during a 4-month period (October 2013 - January 2014). Internal Medicine units were selected with the aim to be representative of this setting on a nationwide basis, by considering geographical distribution, characteristics of the hospital (category, number of beds etc.), and clinical care services. At admission to the IMU information on risk factors for CDI was collected for each patient: age, gender, renal dysfunction (severe: creatinine clearance [CrCl] <30 mL/min; moderate: 30 ≤ CrCl ≤ 65 mL/min), inflammatory bowel disease (IBD), immunosuppression, use of antibiotics, use of proton pump inhibitors, H_2_-receptor antagonists or other antacids, use of laxatives, use of statins, parenteral nutrition, prolonged bed rest (of at least 30 days within 3 months prior to hospitalization), hospitalization (of at least 3-day duration), patient from nursing care home/post-acute care or rehabilitation facilities, previous CDI (within 12 months). Data were recorded on a study-specific electronic case report form, based on contents of the hospital charts. CDI were identified testing all samples of diarrhoea (defined as at least three consecutive episodes selected according to Bristol Stool Chart ≥5) through the same diagnostic test: enzyme immunoassay (EIA) for detecting CD glutamate dehydrogenase (GDH), and A and B toxin (C. Diff Quik Chek® and Tox A/B Quik Chek®, AlereTM). CD diagnosis was considered confirmed if both tests were positive.

**Fig. 1:**
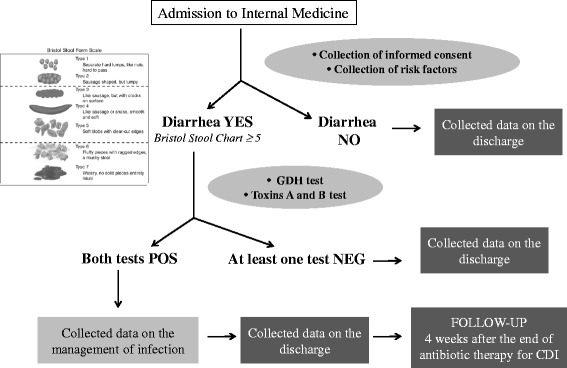
Flow-chart of the study

For patients with diagnosis of CD, information on signs and symptoms of infection, specific treatments, healthcare procedures, complications, recurrences and all-cause mortality was collected. All CDI patients were evaluated during their hospital stay, and a phone follow-up was made 4 weeks after the end of antibiotic therapy for CDI to evaluate recurrences, survival and possible re-hospitalizations (Fig. 1).

Each participating centre received the approval for the study by the local Ethics Committee, and informed consent has been obtained from each enrolled patient.

As a post-hoc central lab evaluation, a real-time Polymerase Chain Reaction (PCR) assay (Xpert® *C. difficile*, Cepheid) was used to evaluate both GDH+/Tox + and GDH+/Tox- samples previously assessed by EIA. This is an in vitro, 45-min real-time PCR assay that provides qualitative detection of toxinogenic strains. The primers and probes in the Xpert *C. difficile* assay detect sequence targets in the genes for Toxin B (*tcd*B), Binary Toxin (*cdt*), and *tcd*C (delection in the toxin A/B negative regulator gene). All stool samples were inoculated on Brucella 5 % SB plate. The cultures from specimens with discordant results between the screening test and the real-time PCR, were re-tested by an alternate EIA method (ImmunoCard® Toxins A&B, Meridian).

*C. difficile* strains were typed using capillary-gel electrophoresis-based PCR-ribotyping according to Indra et al. [21]. Ribotypes were determined by submitting data to the free WEBRIBO database (http://webribo.ages.at) of the Austrian Agency for Health and Food Safety (AGES).

### Statistical analysis

The following common descriptive statistics were calculated: mean with standard deviation (SD) or median with interquartile range (IQR) for continuous variables and absolute and relative frequencies for categorical variables. For continuous variables, statistical comparisons were made using unpaired *t*-test or the analogous non-parametric Wilcoxon Rank Sum test whenever departure from normality distribution was detected by the Shapiro-Wilk test. The Chi-square test or Fisher’s exact test, if deemed more appropriate, were used to analyze categorical variables. Incidence rates were expressed as number of cases/10,000 person-day and reported with 2-sided 95 % Confidence Intervals computed using the Mid-P exact approach based on a Poisson distribution, while 2-sided 95 % Confidence Intervals of prevalence rates were computed using the Mid-P exact approach based on a binomial distribution. Multivariable logistic regression analysis was carried out to evaluate the possible relationship between the occurrence of CDI and the following independent variables: previous CDI (within 12 months); antibiotic use in the previous 4 weeks; previous hospitalization (within 3 months); age (10-year increase); gender; nursing home residents (yes or no); bed resting for at least 30 days; use of proton pump inhibitors; parenteral nutrition (yes or no). These covariates were selected a priori on the basis of their clinical plausibility, and following the general rule of thumb that states that the ratio between the overall number of events and the number of explanatory variables should be at least 10 in order to minimize the risk of overfitting. Results are reported as adjusted Odds Ratios with associated 2-sided 95 % Confidence Intervals. Differences are considered statistical significant for two-tailed *p*-values less than 0.05. The Procedure Logistic of SAS software version 9.4 was used for the multivariable statistical analysis.

## Results

A total of 10,780 patients have been enrolled in the study, with homogenous geographic distribution in Italy. Of these, 5.3 % (95 % CI: 4.9 %–5.7 %) experienced diarrhoea and 0.96 % (95 % CI: 0.78–1.15 %) had diarrhoea caused by CDI (*N* = 103). Symptoms were present in 54 patients at the time of hospital admission (prevalence: 0.52 %, 95 % CI: 0.39–0.67 %). The overall incidence of CDI in the study setting was 4.4/10,000 patient-days (95 % CI: 3.28–5.81). Twenty-nine centers registered at least one case of CDI during the enrolment period, with an average number of 3.5 ± 3.3 CDI patients per center. One center was associated with 18 cases, which was much higher than the other enrolment sites (Table 1).

**Table 1:** Distribution of cases of CDI (*n* = 103) in the participating Centers. At least one case of CDI occurred in 29 Centers; no cases of CDI were detected in 11 Centers

Number of cases of CDI	Code of Center
1	001–003–005–011–013–016-040
2	002–007–008–020–030
3	004–014–015–022–036–038
4	012–023–033–035
5	010–021–031–034
7	009-018
18	029

Patients with CDI were older and more frequently female. More than one-third were in-patients, coming from nursing home (19.4 %), or already hospitalized in other units of the same hospital (17.5 %). Moreover, 58.3 % of patients with CDI had a hospitalization in the previous 3 months *vs* only 23.2 % of patients without CDI (*p* < 0.0001). Overall, 85 % of patients had healthcare-associated CDI (i.e. recent previous hospitalization or nursing home residents or onset of diarrhoea 3 days or more after admission to hospital). A large percentage of CDI patients had a history of prolonged bed rest (40.8 %), and 12.6 % of patients had history of CDI in the previous 12 months (Table 2).

**Table 2:** Baseline characteristics of patients with or without Clostridium difficile infection (CDI). Figures are expressed as number of cases (%) unless otherwise stated. SD = standard deviation. Bed resting = at least 30 days of bed rest within 3 months prior to hospitalization. Immunodepression = systemic corticosteroids/immunosuppressive therapy/HIV infection/active cancer-chemotherapy

Variable	CD infection (*N* = 103)	No-CD group (*N* = 10,677)	*p* value
Age (years, mean ± SD)	80.5 ± 11.0	74.7 ± 14.8	<0.0001
Gender (female)	69 (67.0)	5349 (50.1)	0.0006
Coming from			<0.0001
Home	65 (63.1)	8926 (83.6)	
Nursing-home	20 (19.4)	598 (5.6)	
Another hospital unit	18 (17.5)	1110 (10.4)	
Not assessed	0	43 (0.4)	
Previous hospitalization	60 (58.3)	2477 (23.2)	<0.0001
Bed resting	42 (40.8)	1474 (13.8)	<0.0001
Previous CDI	13 (12.6)	64 (0.6)	<0.0001
Antibiotics treatment (within 4 weeks before)	73 (70.6)	2840 (26.6)	<0.0001
Proton pump inhibitors	75 (72.8)	6193 (58.0)	0.0024
H2-receptor antagonists	1 (1.0)	278 (2.6)	0.3009
Other antacids	4 (3.9)	171 (1.6)	0.0626
Prolonged use of laxatives	9 (8.7)	1014 (9.5)	0.7912
Statins treatment	19 (18.4)	1804 (16.9)	0.6841
Comorbidity – at least 5	37 (35.6)	3459 (32.4)	0.6582
Immunodepression	43 (41.7)	3801 (35.6)	0.1984
Renal failure			0.0055
Mild	30 (29.1)	2530 (23.7)	
Severe	15 (14.6)	801 (7.5)	
No	58 (56.3)	7346 (68.8)	
Inflammatory bowel disease	2 (1.9)	107 (1.0)	0.3232
Parenteral nutrition	5 (4.9)	214 (2.0)	0.0413

About three out of four patients with CDI (70.6 %) had an antibiotic treatment within 4 weeks before diarrhoea *vs* only 26.6 % in the group without CD, and the majority had been treated with a cephalosporin (24.3 %), quinolone (21.9 %) or penicillin (15.8 %). A higher percentage of patients with CDI had mild or severe renal failure (43.7 %) vs. 31.2 % in the group of No-CDI (Table 2).

**Fig. 2:**
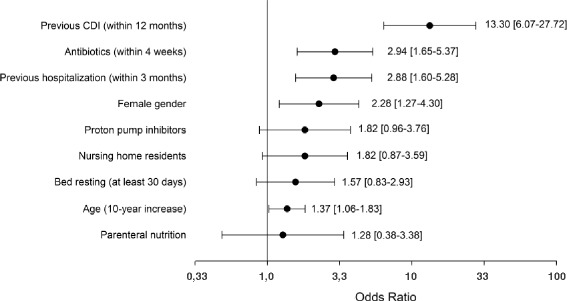
Multivariable analysis to evaluate factors potentially associated with CD infection. Odds Ratios were mutually adjusted for the other variables presented in the Figure. Bars represent the 95 % Confidence Intervals

Multivariable analysis (Fig. 2) demonstrated that previous CDI was the strongest predictor of CDI [OR adjusted 13.30, 95 % CI 6.07–27.72], followed by prior antibiotic treatment [OR adjusted 2.94, 95%CI 1.65–5.37], prior hospitalization [OR adjusted 2.88, 95 % CI 1.60–5.28], female gender [OR adjusted 2.28, 95%CI 1.27–4.30] and age (10-year increase) [OR adjusted 1.37, 95 % CI 1.06–1.83]. Treatment with proton pump inhibitors, nursing home residency, prolonged bed rest and parenteral nutrition did not show significant association, although approaching statistical significance for the first two variables.

In patients with CDI, the therapy of choice was vancomycin (42.8 %), while metronidazole was used in 34 % of patients. In some cases, both treatments were used in the same patient, in combination (11.6 %) or in sequence (11.6 %). Mean duration of CD treatment was 13 days. Probiotics were prescribed in 19.6 % of CDI patients.

Concerning major outcomes, CDI patients showed a doubled length of hospital stay with a median of 16 (IQR = 13) days *vs* 8 (IQR = 8) days in No-CDI (*p* < 0.001) and a greater percentage of in-hospital mortality (crude estimates: 16.5 % *vs* 6.7 %, *p* < 0.001). At least one recurrence within 4 weeks after conclusion of CDI treatment was observed in 14.6 % of patients with 66 % of recurrences occurred in the first 2 weeks of follow-up. Re-hospitalizations during follow-up was 19.8 % and the overall mortality (in-hospital + post-discharge) was 25.2 %.

The post-hoc central lab evaluation showed that CDI was confirmed by molecular method (real-time PCR assay) in 97.3 % of the GDH+/Tox + samples. On the other hand, the PCR assay detected positive samples for the toxin in 23 out 34 (67.6 %) cases which were GDH+/Tox- by EIA. By adding incident cases within this subgroup, CDI incidence rate was estimated to be 5.3/10.000 patient-days (4.09–6.88).

**Fig. 3:**
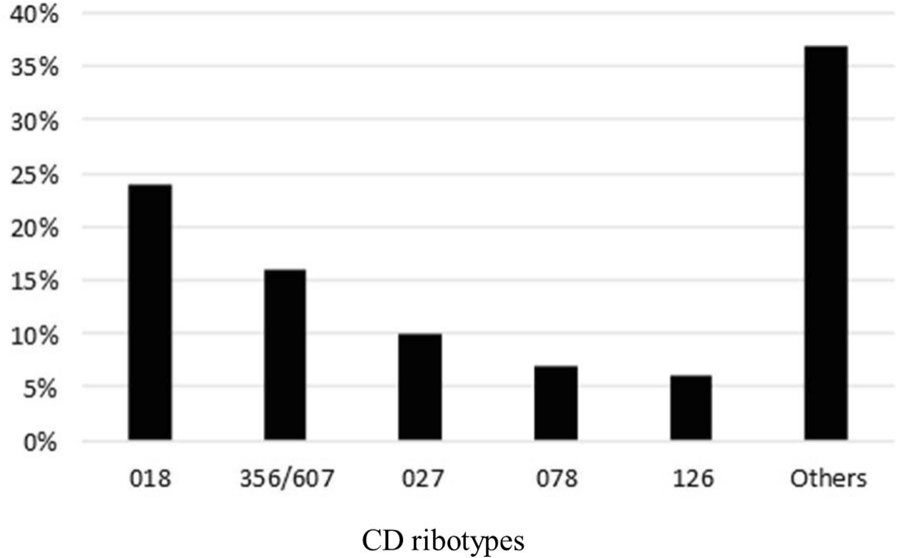
Distribution of CD ribotypes identified in the study population

We identified 26 different ribotypes of *C. difficile* among the 70 strains that were subjected to ribotyping. The predominant ribotype was 018 (24.3 %), followed by 356/607 (15.6 %), 027 (10 %), 078 (7.1 %) and 126 (5.6 %) (Fig. 3). Ribotype 356/607 is identified as 356 or 607 by the University of Leeds database (UK) and the Austrian Agency for Health and Food Safety (AGES) database, respectively [22]. One cluster of infection related to ribotype 018 was detected in the centre with the highest number of CDI cases (see Table 1). In-hospital mortality rates related to specific CD ribotypes were 11.7, 9.0, 14.3 and 0 % for 018–356/607–027 and 078 ribotypes, respectively.

## Discussion

Our results confirm that CDI are a not negligible finding (incidence: 5.3/10,000 patient-days) among patients hospitalized in IMU in Italy. Although direct comparison with previously published Italian data (reporting incidence rates from 0.3 to 22.3/10,000 patient-days) is difficult due to heterogeneity of study designs, timing of evaluation, and settings, our estimate of incidence rate is within the range reported for Italy in two recently published European surveys [16, 19]. In our survey, CDI accounted for 1 out of 5 cases of diarrhoea among inpatients. When both incident and prevalent cases are considered, around 1 % of all patients hospitalized in Italian IMU had diagnosis of CDI (around 1.5 million patients are admitted to IMU in Italy per year). Finally, healthcare-associated CDI (recent previous hospitalization/nursing home residents/onset of diarrhoea 3 days or more after admission to hospital) accounted for the majority of cases, but a sizeable percentage of CDI was community-acquired (15 %). Substantial rates of CDI in the community are probably related to a number of factors [23], and have been recently reported in the literature [24].

As a major finding from our study is that CDI seems associated with a worse outcome, with a three-time higher in-hospital overall mortality *vs* no-CDI patients (16.5 % *vs* 6.7 %), and in nearly half of cases (46 %) CDI was considered related to patient death. Historically, the attributable mortality of CDI was considered low (less than 2 % of cases) [25]. However, consistent with our findings, more recent literature has reported a marked increase of CD-associated mortality and case-fatality rates [11, 26, 27]. In addition, in our survey CDI was associated with a significantly longer (doubled) hospital stay, thus confirming the high economic burden of this infection [28].

Recurrence is one of the most important challenges in the management of CDI. Our period of observation (4 weeks after completion of antibiotic treatment for CDI and actually around 6 weeks after diagnosis of CDI) is somewhat shorter but quite similar to that indicated by international guidelines to define the presence of recurrence (8 weeks after the onset of a previous episode of CDI) [2]. In our study, recurrence occurred in around 15 % of patients, mostly during the first 2 weeks of follow-up. This recurrence rate is slightly lower than some previous reports [9, 10], but still clinically relevant; in this perspective, it will be interesting to see in the future the impact, in real-life, of novel treatments for CDI which have been associated with a lower risk of recurrence [10, 29, 30].

Most of the predisposing factors we found as significantly related to CDI (positive history for the infection, use of antibiotics, recent hospitalization, and age), are consistent with those reported in previous studies [16]. In particular, antibiotic therapy is confirmed as a significant predictor of CDI, though around one-third of our patients affected with CD had a negative history for antibiotics. Our data suggest that clinical suspicion of diarrhoea from CDI should be driven not solely by the presence of a current or recent antibiotic course. This point is especially important in outpatients [31], who may be at greater risk of underdiagnosis. Another unique finding of our study was that females appeared at increased risk of CDI, which has not been previously reported [32], and required future confirmation. A trend towards higher incidence of CDI has been observed in our study for patients receiving proton-pump inhibitors. This item has been addressed in several studies, and our data seem to support the results of recent meta-analyses showing a 50 to 65 % increase in the incidence of CDI among patients on proton-pump inhibitors [33, 34]. However, concerns have been raised on the quality evidence of this association [35, 36], and further studies, preferably prospective, are needed to fully explore the causative relationship between proton-pump inhibitors and CD-associated diarrhoea. A limitation of our study was that we were not able to explore predictors of CDI recurrence due to the relatively low number of cases with recurrent CDI in our cohort.

A rapid and accurate laboratory diagnosis is crucial for optimizing the prevention and management of CDI, and many different approaches are available. However, the most appropriate approach for diagnosis remains a topic of debate, and very recent findings raised concern on possible overdiagnosis rather than on under-recognition of the infection [37]. Preferably a two- or three-stage algorithm should be performed to diagnose CDI, in which a positive first test is confirmed with one or two confirmatory tests or a reference method [2, 3]. In our experience, the two-step EIA method for detection of *C.difficile* GDH and A and B toxins, showed a very high specificity (more than 95 %); however, the percentage of false negative for toxins among patients with positive GDH was substantial, and largely corrected by a molecular method (the real-time PCR assay Xpert® *C. difficile*). Our data support the value of a two-step algorithm including EIA GDH and toxins when both tests are positive, and an additional molecular test (or toxigenic culture) for toxins in case of GDH+/TOX– samples by EIA.

The majority of strains isolated in this study belonged to ribotype 018 or 356/607. These ribotypes are predominant in Italy and are phylogenetically related [22, 38]. In particular, infection by ribotype 018 is associated with complicated CDI [16]. In the last several years, hypervirulent strains 027 and 078 have become more prevalent causes of CDI in Italy [22, 38–40]. In our study, none of the ribotypes showed a significant correlation with adverse outcome, probably due to the low number of samples.

One potential strength of the FADOI-PRACTICE study is its prospective design. This allowed a reliable assessment of associated independent variables for CDI, as well as a strict follow-up in patients with CD-associated diarrhoea. Further, it was possible to systematically apply (in all cases with diarrhoea) the same approach for laboratory diagnosis of CDI, along with a post-hoc assessment for all GDH positive samples; together with the rigorous method for selection of participating centers and the screening of the total population of all patients admitted to IMUs, this reasonably makes our findings of interest and accurate for the specific study setting. On the other hand, as a possible limitation of our study, the 4-month enrolment period may have obscured possible seasonal variations in the occurrence of CDI, as previously reported [18]. In addition, no follow-up was scheduled for patients with GDH+/Tox- samples, and since some of these patients were considered positive for CDI by the PCR method, this could make our outcome results not complete.

## Conclusions

In conclusion, data from the FADOI-PRACTICE study demonstrate that, on a nationwide basis, CDI is worth considering and a potentially severe complication among patients hospitalized in Italian Internal Medicine wards (at least one case was detected in 29 out of the 40 participating centers). This observation should lead clinicians to suspect CDI in presence of diarrhoea and risk factors, and makes improvements in the surveillance systems advisable. Clinicians should be aware of the diagnostic algorithms, including their possible limitations, utilized in the clinical microbiology laboratory of their hospital. In this perspective, and based on a strict cooperation between clinicians and clinical microbiologists, our survey supports the use of a 2–3 step algorithm for diagnosis: enzyme immunoassay for detecting *C.difficile* GDH and A/B toxin, and a molecular method (e.g. a real-time PCR assay) in case of toxin negative samples.

## Additional file

Additional file 1: List of local Ethics Committees which approved the study. (DOC 25 kb)

## Abbreviations

CD: Clostridium difficile
CDI: Clostridium difficile infection
CrCl: Creatinine clearance
EIA: Enzyme immunoassay
GDH: Glutamate dehydrogenase
IBD: Inflammatory bowel disease
IM: Internal medicine
OR: Odds ratio
PCR: Polymerase Chain Reaction
Tox: Toxin
