# Supplementary material for: Epidemiology and outcome of Clostridium difficile infections in patients hospitalized in Internal Medicine: findings from the nationwide FADOI-PRACTICE study
Source: BMC Infect Dis. 2016 Nov 8;16:656. doi: 10.1186/s12879-016-1961-9 (PMC5101712; doi:10.1186/s12879-016-1961-9)
Supplement: Additional file 1: — List of local Ethics Committees which approved the study. (DOC 25 kb) [file 12879_2016_1961_MOESM1_ESM.doc]

Manuscript

**Epidemiology and outcome of *Clostridium difficile* infections in patients hospitalized in Internal Medicine. Findings from the nationwide FADOI-PRACTICE study**

by G. Cioni et al.

**Additional File – List of Ethics Committees which approved the study**

Comitato Etico dell'Ospedale Civile di Legnano (MI) - Coordinating Centre

Comitato Etico Regionale AO "Ospedali Riuniti" di Ancona

Comitato Etico Ospedale "S. Donato" di Arezzo

Comitato Etico dell'Ospedale Maggiore di Bologna

Comitato Etico dell'ASL di Cagliari

Comitato Etico della Provincia di Padova

Comitato Etico ARNAS "Garibaldi" di Catania

Comitato Etico dell'Ospedale "Pugliese-Ciaccio" di Catanzaro

Comitato Etico dell'ASL di Foggia

Comitato Etico ASL Roma G

Comitato Etico della Provincia di Treviso

Comitato Etico dell'Ospedale "Maggiore della Carità" di Novara

Comitato Etico della Provincia di Ferrara

Comitato Etico dell'AUSL di Piacenza

Comitato Etico dell'AOU "Careggi" di Firenze

Comitato Etico dell'ASF di Firenze

Comitato Etico dell'Azienda Sanitaria Regionale del Molise

Comitato Etico dell'Ospedale "Niguarda" di Milano

Comitato Etico dell'AO "Cardarelli" di Napoli

Comitato Etico dell'Ospedale dell'Ente Ecclesiastico "F. Miulli" di Acquaviva delle Fonti (BA)

Comitato Etico della Provincia Romana di S. Pietro "Fatebenefratelli" di Roma

Comitato Etico della Provincia di Modena

Comitato Etico dell'AUSL 3 di Pistoia

Comitato Etico dell'ASL 2 Savonese

Comitato Etico dell'AOU Pisana - Pisa

Comitato Etico del Policlinico "Gemelli" di Roma

Comitato Etico dell'Ospedale "S. Giovanni Calibita - Fatebenefratelli" di Roma

Comitato Etico dell'Ospedale "S. Camillo" di Roma

Comitato Etico della Provincia di Vicenza

Comitato Etico della Provincia di Padova

Comitato Etico Regionale dell'Umbria

Comitato Etico dell'ASL TO 2 di Torino

Comitato Etico Indipendente dell'Azienda Ospedaliera della Provincia di Pavia
